# Spontaneous Pregnancy in Genetically Confirmed 11-Beta Hydroxylase Deficiency: A Case Series and Literature Review

**DOI:** 10.7759/cureus.94504

**Published:** 2025-10-13

**Authors:** Pushpa Machineni, Asha Ranjan, Adlyne Reena Asirvatham, Kunal Gupta, Shriraam Mahadevan

**Affiliations:** 1 Endocrinology, Diabetes and Metabolism, Sri Ramachandra Institute of Higher Education and Research, Chennai, IND

**Keywords:** 11-beta-hydroxylase deficiency, congenital adrenal hyperplasia, cyp11b1 gene, fertility, hypokalemia, nephrocalcinosis, pregnancy

## Abstract

11-beta hydroxylase deficiency (11βOHD) is a rare variant of congenital adrenal hyperplasia (CAH) with autosomal recessive inheritance, resulting in androgen excess and mineralocorticoid precursor accumulation. Fertility is often impaired due to hyperandrogenism and anatomical abnormalities, and spontaneous successful pregnancy in classic form is rare. We describe two cases. Case 1 is a 27-year-old woman with genetically confirmed classic 11βOHD (homozygous CYP11B1 splice-site variant c.240-2A>G), initially misdiagnosed as 21-hydroxylase deficiency, who achieved spontaneous conception despite high androgen levels, long-term steroid exposure, and prior genital surgery and delivered a healthy male child. Case 2 is a genetically confirmed non-classic 11βOHD (homozygous, missense variation, c.412C>T), presented with medullary nephrocalcinosis, clinical history of polycystic ovary syndrome (PCOS) in adolescence, short stature, hypertension with hypokalemia, and had spontaneous pregnancies. These cases add to the very limited literature on spontaneous fertility in genetically confirmed 11βOHD cases and highlight its broad clinical spectrum. They emphasize the need for accurate diagnosis, long-term complications surveillance, and reproductive counselling in CAH patients.

## Introduction

11-beta hydroxylase deficiency (11βOHD) is a rare variant of congenital adrenal hyperplasia (CAH), resulting from mutations in the CYP11B1 gene. The enzymatic defect leads to the accumulation of 11-deoxycortisol and deoxycorticosterone, and excess production of adrenal androgens. Clinically, it is characterized by hypertension, virilization, and glucocorticoid deficiency. The classic form is typically present in infancy with virilization in female infants, and the non-classic form manifests later in childhood or adolescence with signs of androgen excess such as hirsutism and oligomenorrhea in females [1]. Impaired fertility may be due to both hormonal and anatomical factors. Elevated adrenal androgens impair folliculogenesis, ovulation, and endometrial receptivity. Concurrently, increased adrenal progesterone secretion alters gonadotropin-releasing hormone (GnRH) and luteinizing hormone (LH) pulsatility, impairs cervical mucus quality, and disrupts endometrial development and implantation. Structural abnormalities from prior genital reconstructive surgeries may further compromise reproductive potential [2].

## Case presentation

Case 1 is a 27-year-old female, born of a second-degree consanguineous marriage, who had ambiguous genitalia at birth with no hyperpigmentation and adrenal crisis. Clinical examination revealed clitoromegaly, fused labia minora with a single urogenital opening-Prader stage III. Karyotype was 46, XX. Biochemical evaluation revealed elevated 17-hydroxyprogesterone (Table 1). Pelvic ultrasound showed a normal uterus with no adrenal hyperplasia. A presumptive diagnosis of 21-hydroxylase deficiency was made, and hydrocortisone was initiated. Fludrocortisone was added subsequently. At six months of age, the patient underwent recession clitoroplasty and vaginoplasty. She attained menarche at age 15 with normal secondary sexual characteristics and had regular menstrual cycles. However, she had persistent hypokalemia with no history of hypertension, and at the age of 17, after discontinuing glucocorticoid therapy for re-evaluation, she developed an episode of hypokalemic paralysis. Biochemical investigation at that time revealed elevated 11-deoxycortisol (Table 1), and dexamethasone was restarted. She was later diagnosed with hypertension, which was well controlled with glucocorticoid therapy. During the course, her androgen levels were well controlled. Genetic testing confirmed CYP11B1 homozygous splice-site variant mutation (c.240-2A>G), establishing the diagnosis of classic 11β-OHD. She conceived spontaneously; during pregnancy, she was shifted to prednisolone 7.5mg per day. She had an uneventful antenatal course (no gestational diabetes mellitus (GDM)) and delivered a healthy male child.

**Table 1:** Laboratory results – Case 1 (classic 11-beta hydroxylase) and Case 2 (non-classic 11-beta hydroxylase) ^a^ Performed with liquid chromatography-tandem mass spectrometry (LC-MS/MS) 11β-OHD: 11-beta-hydroxylase deficiency.

Hormone/Test	Case 1 Result (Reference range)	Case 2 Result (Reference range)
Adrenocorticotropic hormone (ACTH) (pmol/L)	—	74 (<10.1)
Cortisol (nmol/L)	109(132–537)	219 (132–537)
17-Hydroxyprogesterone (nmol/L)	28.7(0.6-3.9)	249.3 (ACTH Stimulated) (6–66.5)^a^
11-Deoxycortisol (nmol/L)	41.5(<3.1)	546.1 (1.5-8.7)^a ^
11-Deoxycorticosterone (nmol/L)	—	64.1 (0.06-0.44)^a^
Androstenedione (nmol/L)	—	63 (1.04-12.2)^a^
Total Testosterone(nmol/L)	12.1(0.7-2.8)	8.4(0.7-2.8)^a^
Dehydroepiandrosterone sulfate(DHEAS) (µmol/L)	0.49(1.6-9.1)	15.98(1.6-9.1)
Sodium (mEq/L)	138(135–145)	141 (135–145)
potassium (mEq/L)	3 (3.5–5.0)	2.9 (3.5–5.0)
Bicarbonate(mEq/L)	21(21-31)	22(21-31)
Chloride(mEq/L)	101(101-109)	103(101-109)
Calcium (mmol/L)	—	2.35(2.15-2.50)
Phosphorus (mmol/L)	—	1.29 (0.81-1.45)
Vitamin D (nmol/L)	—	55 (>50)
Parathyroid hormone (pmol/L)	—	3.6(1.6-6.9)
Creatinine(µmol/L)	52(44 – 80)	60(44 – 80)

**Figure 1:**
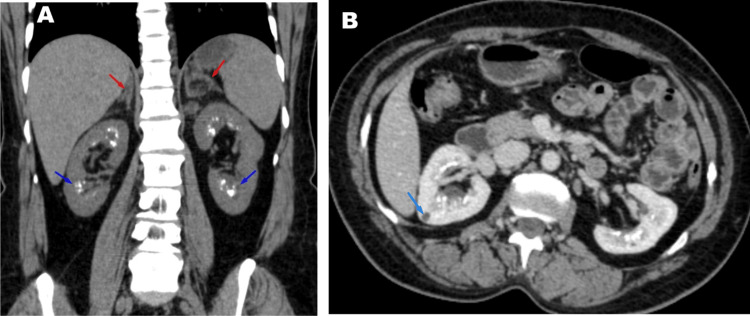
CT scan findings of Case 2: non-classic 11-beta hydroxylase deficiency A: Bilateral adrenal hyperplasia with myelolipoma (red arrow). Medullary nephrocalcinosis (blue arrow) B: Renal cyst with medullary nephrocalcinosis

Case 2 is a 37-year-old female, born of a second-degree consanguineous marriage, who had normal female genitalia at birth, with a positive family history of 11β-OHD in her nephews. At age 10, she developed hyperpigmentation, hirsutism, and menstrual irregularities but was not evaluated. Later, she developed hypertension and hypokalemia, which remained unevaluated for many years. She had two spontaneous, uncomplicated pregnancies, delivered one female and one male child. A Computed Tomography (CT) scan done later for unrelated reasons revealed bilateral adrenal hyperplasia and medullary nephrocalcinosis with renal cysts (Figure 1A, 1B). On examination, she had short stature (height: 140 cm, -3.2 SDS), hirsutism, clitoromegaly, and hyperpigmentation. With the above findings, CAH was suspected and evaluated further. Biochemical and steroid profiling are presented in Table 1. Steroid profiling revealed elevated 17-hydroxyprogesterone and 11-deoxycortisol (546.1 (1.5-8.7)), consistent with the diagnosis of CAH. Evaluation for medullary nephrocalcinosis revealed normal serum calcium, phosphorus, parathyroid hormone, and urinary calcium levels, with no evidence of metabolic acidosis. Genetic testing revealed a homozygous missense variant mutation in CYP11B1: c.412C>T (p. Arg138Cys), confirming the diagnosis of non-classic 11β-OHD. She was initiated on dexamethasone, following which her androgen levels showed a progressive decline, and menstrual cycles normalized over subsequent months. Blood pressure remained within the normal range on follow-up without the need for additional antihypertensive agents.

## Discussion

CAH is most commonly caused by 21-hydroxylase deficiency, while 11β-OHD accounts for approximately 5-8% of cases and has an estimated incidence of one in 100,000 live births [3]. This condition results from mutations in the CYP11B1 gene, leading to defective conversion of 11-deoxycortisol and deoxycorticosterone to cortisol and corticosterone, respectively. The biochemical consequences include cortisol deficiency, accumulation of 11-deoxycorticosterone (a potent mineralocorticoid), elevated 11-deoxycortisol, and excess adrenal androgens. Clinically, this manifests as hypertension (with suppressed renin), hypokalemia, hyperandrogenism, and genital ambiguity. Females with the classic form typically present at birth with ambiguous external genitalia and normal internal reproductive anatomy, often requiring early surgical correction. Phenotypic variability depends on residual enzymatic activity [4]. Our patient with the classic form of 11β-OHD presented in infancy with ambiguous genitalia consistent with in utero androgen excess. In our case, initial elevation of 17-hydroxyprogesterone led to a presumptive diagnosis of 21-hydroxylase deficiency, a common diagnostic pitfall due to overlapping features with 11β-OHD. Upon withdrawal of glucocorticoids, the patient experienced an episode of hypokalemic paralysis, which was likely secondary to unopposed deoxycorticosterone-mediated mineralocorticoid activity. Though elevated 17-hydroxyprogesterone levels most commonly suggest 21-hydroxylase deficiency, it is crucial to consider less common conditions such as 11β-OHD in the differential diagnosis of CAH. Similar clinical courses have been reported in the literature [5]. Hypertension and hypokalemia are characteristic features of 11β-OHD, distinguishing it from 21-hydroxylase deficiency, which typically presents with hypotension and hyperkalemia. However, the markedly elevated 11-deoxycortisol level and the identification of a homozygous CYP11B1 splice-site mutation (c.240-2A>G) ultimately confirmed 11β-OHD in this case. This diagnostic delay highlights the importance of specific steroid precursor profiling in all cases of CAH, particularly when hypertension or hypokalemia is present. Our patient had normal secondary sexual characteristics and regular menstrual cycles while on appropriate glucocorticoid replacement, suggesting adequate adrenal androgen control. Despite the well-known challenges to fertility in 11β-OHD, owing to elevated follicular-phase progesterone, gonadotropin suppression, and anatomical alterations, she achieved spontaneous conception. She was well maintained on prednisolone throughout pregnancy without any hypertensive events. This case emphasizes the potential for fertility in classic 11β-OHD when managed appropriately. Only a few genetically confirmed severe classic forms of 11β-OHD cases with successful pregnancy have been reported. A comparison with the literature (Table 2) reveals further heterogeneity. Simm et al. [6] reported a patient requiring ovulation induction with clomiphene, hyperandrogenism, and intensified glucocorticoid therapy during pregnancy. Kavitha et al. [7] described a patient on prednisolone and antihypertensives whose pregnancy was complicated by severe preeclampsia, necessitating emergency cesarean at 33 weeks. Demir et al. [8] also reported spontaneous conception following multiple reconstructive surgeries, with glucocorticoid adjustment during pregnancy. In pregnant women with 11β-OHD, hypertension is managed with glucocorticoids (hydrocortisone or prednisolone) to suppress adrenocorticotropic hormone (ACTH) driven mineralocorticoid excess. If additional therapy is needed, pregnancy-safe antihypertensives such as labetalol or nifedipine may be added, while angiotensin-converting enzyme inhibitors, angiotensin receptor II blockers, and thiazides are avoided [3,9]. During pregnancy, dexamethasone should be avoided because placental 11β-hydroxysteroid dehydrogenase type 2 does not inactivate it, allowing significant transplacental passage; and stress dose glucocorticoids are recommended during labour [10]. Cesarean section was required in 52-84% of cases, primarily due to a history of genital reconstructive surgery. Although GDM is a recognized complication in CAH pregnancies, our patient did not develop GDM. Given the limited literature on pregnancies in women with 11β-hydroxylase deficiency, clinical management is mostly guided by experience from the more prevalent 21-hydroxylase deficiency [4,11].

**Table 2:** Comparison with literature. NR: not reported; GHTN: gestational hypertension; 11β-OHD: 11-beta-hydroxylase deficiency

Parameter	Classic 11β-OHD				Non-classic 11β-OHD
	Case 1	Simm et al., 2007 [6]	Kavitha et al., 2023 [7]	Demir et al., 2021 [8]	Case 2
Genetic Mutation	Homozygous c.240-2A>G	Heterozygous intron DS+2 splicing + exon 8(G444D)	NR	Homozygous p.L299P (c.896T>C)	Homozygous c.412C>T
Clinical Presentation	Ambiguous genitalia at birth	Ambiguous genitalia at birth	Ambiguous genitalia	Ambiguous genitalia at birth	PCOS-like, short stature, Hypertension with hypokalemia
Surgical History	Clitoroplasty + vaginoplasty	Clitoral reduction + introitoplasties.	Clitoral resection + vaginoplasty + dilation	Surgeries at 4 and 24 years	None
Hypertension	Yes	No	Yes	Yes	Yes
Mode of Conception	Spontaneous	Clomiphene-induced	NR	Spontaneous	Spontaneous
Steroid Regimen	Prednisolone 7.5mg	Dexamethasone ↑ to 2 mg/day	Prednisolone	Methylprednisolone + Dexamethasone → Hydrocortisone	None (prenatal/natal)
Pregnancy Outcome	Uneventful. Elective C-section at 38weeks.Male child.	GHTN. Hyperandrogenism. Elective C-section at 37 weeks. Male child.	Severe preeclampsia. Emergency C-section at 33 weeks. Male child.	Elective C-section at 34 weeks. Female child.	Uneventful. Elective C-section at 37 weeks, Male and female child

In contrast, the non-classic 11β-OHD patient had a much subtler phenotype and was diagnosed in adulthood despite early signs of hyperandrogenism, short stature, and menstrual irregularities from childhood. The normal appearance of external genitalia at birth and lack of salt-wasting symptoms delayed recognition, as is frequently seen in non-classic 11β-OHD. These patients are often misdiagnosed with polycystic ovary syndrome (PCOS) [4,12,13], and diagnosis requires a high index of suspicion, especially when accompanied by persistent hypertension and unexplained hypokalemia. In this case, significantly elevated levels of 11-deoxycortisol and deoxycorticosterone, along with ACTH elevation and genetic confirmation of a homozygous missense mutation in CYP11B1 (c.412C>T; p. Arg138Cys), were diagnostic. Imaging revealed bilateral adrenal hyperplasia, consistent with the underlying enzymatic defect. Additionally, she was found to have renal cysts and medullary nephrocalcinosis, most likely a consequence of long-standing chronic hypokalemia. Similar renal manifestations in patients with 11β-OHD have been previously reported by Aswani et al. and Abdulla et al. [14,15]. Remarkably, she achieved spontaneous pregnancies despite the absence of treatment, highlighting the broad clinical variability of non-classic 11β-OHD. Timely recognition and genetic confirmation are critical to prevent long-term complications and optimize reproductive outcomes.

## Conclusions

These cases illustrate the broad clinical spectrum of 11β-OHD and the importance of distinguishing it from 21-hydroxylase deficiency through 11-deoxycortisol (and deoxycorticosterone when possible) measurement and genetic confirmation. Hypertension and hypokalemia are key diagnostic clues, and long-term complications like nephrocalcinosis require surveillance. Fertility is achievable with appropriate glucocorticoid therapy and management of mineralocorticoid and androgen excess. Increased awareness of the non-classic presentation is crucial to prevent diagnostic delays and associated morbidity.
